# Gender disparities in ophthalmology academic conferences in Japan

**DOI:** 10.1097/MD.0000000000043057

**Published:** 2025-07-04

**Authors:** Hiromi Onouchi, Akemi Iwasaki, Yuka Morita, Mariko Itakura, Keiko Kunimi, Yoichi Manabe, Naoko Kato

**Affiliations:** a Department of Ophthalmology, International University Health and Welfare, Atami Hospital, Shizuoka, Japan; b Otaki Eye Clinic, Chiba, Japan; c Department of Ophthalmology, Tsukuba University Hospital, Ibaraki, Japan; d Maebashi Minami Eye Clinic, Gumma, Japan; e Department of Ophthalmology, Kitasato University School of Medicine, Kanagawa, Japan; f Minamiaoyama Eye Clinic, Tokyo, Japan.

**Keywords:** diversity, domestic congress, first presenter, gender disparity, moderator

## Abstract

Despite a rise in the number of female physicians, their underrepresentation persists. This disparity grows with increasing academic rank, resulting in the loss of professionals within academic medical institutions. The purpose of the present study is to investigate gender disparity in the Academy of Ophthalmology in Japan. A retrospective observational study was conducted. The number of the first presenters and moderators in 24 of the Japanese academic congresses held in 2023 was assessed. Data of the first presenters and moderators were collected from the website or abstract book of each congress. Gender and the nationality (Japanese or non-Japanese) of the first presenters and moderators were estimated from their given names and photographs. The study comprised 1325 (25.2%) women and 3933 (74.8%) men across all congresses. Among them, 1116 (27.2%) of the first presenters and 209 (18.0%) of the moderators were women. When the sessions were classified into 4 categories as nominated/guest/special lectures, award lectures, symposium/instruction/educational lectures, and free paper/posters, female representation was 24.5%, 20.7%, 20.9%, and 31.4% among first presenters, and 17.0%, 11.4%, 16.3%, and 21.6% among moderators, respectively. The percentage of non-Japanese first presenters was 1.3% in women and 1.4% in men, and 2.4% and 0.0% in the moderators, respectively. The participation rate of women and non-Japanese individuals in the academic congresses of ophthalmology in Japan is remarkably low. In contrast, Japanese men account for the majority of participants, highlighting a significant imbalance. This suggests a lack of diversity within the society’s membership.

## 1. Introduction

The number of female physicians has been increasing. In many industrialized countries, it is reported that female physicians account for 50% of all physicians.^[1,2]^ Despite a rise in the number of female physicians, their underrepresentation, particularly in leadership roles, persists even in Western countries.^[3]^ This disparity grows with increasing academic rank, resulting in the loss of professionals within academic medical institutions. Women remain underrepresented in chairs, professorships, and council memberships within medical faculties, academic societies, and medical journal editorial boards.^[3,4–11]^

Several previous investigations revealed that female physicians’ publication indices, both quantitative and qualitative, lag those of their male counterparts.^[11–16]^ The number of high-quality academic publications may best reflect the level of academic activity. However, the experiences of the first presenters in congresses also form a key component in evaluating one’s scholarly capabilities. First presenter experience in congresses and publications authored by early-career researchers indicates their level of activity. Furthermore, participation as a moderator or committee member in academic congresses indicates a leading role in their respective fields of study.

In the field of ophthalmology, it has been reported that the representation of women in academic medicine is still lower than men, especially in the subspecialties related to surgery.^[17–19]^ Published studies indicate a substantially greater number of publications by men compared to women.^[20]^ Among authorship, the percentage of women last authorship is reported to be less than that of the women first authorship.^[17,18]^ Compared to men, women are underrepresented as professors, department chairs, division chairs, and editorial board members of academic journals.^[17]^ The proportion of female presenters at academic conferences and meetings was found to be lower than the proportion of female members in academic societies.^[18]^ Furthermore, a higher proportion of female participants self-submitted their presentations compared to those who were invited.^[21,22]^

The faculties of ophthalmology, dermatology, gynecology, and pediatrics in Japan are characterized by a substantially higher percentage of female physicians than in other fields. Our previous study^[23]^ revealed a lower representation of women in academic ophthalmology departments at Japanese medical schools. The underrepresentation of women can lead to a less suitable working environment for them, which in turn may widen the gap between female and male ophthalmologists. Such discrepancies may result in a higher resignation rate among female ophthalmologists, even though their proportion is increasing. This may ultimately lead to a shortage of ophthalmologists, and hinder the uptake of cutting-edge knowledge and technologies among ophthalmologists in Japan.

To our knowledge, no subsequent research has addressed female ophthalmologist representation in academia. Therefore, we conducted this study to analyze the gender disparity among the first presenters and the moderators in the domestic ophthalmology congresses in Japan.

## 2. Methods

We conducted a comprehensive survey assessing the gender and nationality of all first authors and moderators of the 24 domestic academic congresses held in Japan in 2023. First author and moderator names were compiled from the online programs of twenty-four congresses and the abstract book of 1 congress, as its program was unavailable online. Presenter gender and nationality were determined from given names and photographs, where available.

Statistical analyses were performed using JMP12 software (SAS Institute Inc., Cary). We considered *P* values < .05 as statistically significant. The chi-square test was used to evaluate differences in the percentage of female and non-Japanese physicians in the first presenters and moderators among the 4 types of lectures. *P* < .05 was considered statistically significant.

## 3. Results

There were 5339 presenters (including first presenters and the moderators) across 24 ophthalmology congresses. The nationality and gender of 81 presenters could not be determined, so they were excluded. Thus, the study encompassed 5258 presenters comprising 1325 women (25.2%) and 3933 men (74.8%). The number of presenters with supposed Japanese nationality was 5198 (98.9%) and those with supposed non-Japanese nationality was 60 (1.1%).

Among the first presenters, 27.2% (1116 of 4098) were women. The percentage of women was varied among the congresses (Table 1); the congress with the highest percentage of female presenters was Japanese Association for Strabismus and Amblyopia/Japanese Association for Pediatric Ophthalmology (JASA/JAPO, 58.7%), then, followed by Japan Society of Low-vision Research and Rehabilitation (JSLRR, 49.4%), Japan Imaging and Perimetry Society (JIPS, 38.1%), Japan Cornea Conference (JCC, 34.5%), and Japanese Society of Ophthalmic Plastic and Reconstructive Surgery (32.5%). Conversely, Japanese Society of Ocular Oncology exhibited the lowest proportion of female members (14.6%), followed by Japanese Society of Lacrimal Passage and Tear Dynamics (JSLT, 15.4%), Japanese Society of Ophthalmic Diabetology (16.5%), Japanese Society of Ophthalmic Surgery (18.0%), and Japanese Society of Cataract and Refractive Surgery (JSCRS, 18.9%).

**Table 1:** Gender balance of the first presenters in Japanese domestic congresses for ophthalmology.

	JSOS	JCC	JOS	JMS	JIPS	JSOPRS	JASA/JAPO	JSCRS	JSOD	JSLRR	JSOOp	JCLS	JOIS	JAOI
Women	64	68	157	16	16	13	74	55	14	40	12	14	17	14
Men	292	129	504	43	26	27	52	236	71	41	28	32	37	36
Women (%)	18.0	34.5	23.8	27.1	38.1	32.5	58.7	18.9	16.5	49.4	30.0	30.4	31.5	28.0

	JSLT	Foursome-joint*	JSCR	JSOC	JSOOn	JGS	JCO	JSOP	JSAIO	JRVS	JNOS	Total		
Women	4	3	21	30	6	75	305	14	7	51	26	1116		
Men	22	6	54	80	35	178	739	32	25	184	73	2982		
Women (%)	15.4	33.3	28.0	27.3	14.6	29.6	29.2	30.4	21.9	21.7	26.3	27.2		

JAOI = Japanese Association for Ocular Infection, JASA/JAPO = Japanese Association for Strabismus and Amblyopia/Japanese Association for Pediatric Ophthalmology, JCC = Japan Cornea Conference, JCLS = Japan Contact Lens Society, JCO = Japan Clinical Ophthalmology, JGS = Japan Glaucoma Society, JIPS = Japan Imaging and Perimetry Society, JMS = Japan Myopia Society, JNOS = The Japanese Neuro-Ophthalmology Society, JOIS = Japanese Ocular Inflammation Society, JOS = Japan Ophthalmological Society, JRVS = Japanese Retina and Vitreous Society, JSAIO = Japanese Society of Artificial Intelligence in Ophthalmology, JSCR = Japanese Society for Cataract Research, JSCRS = Japanese Society of Cataract and Refractive Surgery, JSLRR = Japan Society of Low-vision Research and Rehabilitation, JSLT = Japanese Society of Lacrimal Passage and Tear Dynamics, JSOC = Japanese Society for Ocular Circulation, JSOD = The Japanese Society of Ophthalmic Diabetology, JSOOn = Japanese Society of Ocular Oncology, JSOOp = Japanese Society of Ophthalmological Optics, JSOP = Japanese Society for Ocular Pharmacology, JSOPRS = Japanese Society of Ophthalmic Plastic and Reconstructive Surgery, JSOS = Japanese Society of Ophthalmic Surgery.

* Joint lecture of JCLS, JOIS, JAOI, and JSLT.

Among the moderators, 18.0 % (209 out of 1160; Table 2) were women. Japanese Association for Ocular Infection (38.5%) exhibited the highest proportion of female members followed by JSLRR (37.9%), Japan Clinical Ophthalmology (36.0%), JCC (28.0%), and JASA/JAPO (23.3%). Conversely, the congresses exhibiting the lowest proportions of female representation were the Japanese Neuro-Ophthalmology Society (7.3%), followed by JSCRS (7.9%), Japanese Society of Artificial Intelligence in Ophthalmology (8.3%), JSLT (10.0%), and JIPS (10.5%).

**Table 2:** Gender balance of moderators in the Japanese domestic congresses for ophthalmology.

	JSOS	JCC	JOS	JMS	JIPS	JSOPRS	JASA/JAPO	JSCRS	JSOD	JSLRR	JSOOp	JCLS	JOIS	JAOI
Women	21	7	33	4	2	2	7	9	4	11	3	4	2	5
Men	135	18	170	21	17	14	23	105	24	18	14	14	16	8
Women (%)	13.5	28.0	16.3	16.0	10.5	12.5	23.3	7.9	14.3	37.9	17.6	22.2	11.1	38.5

	JSLT	Foursome-joint*	JSCR	JSOC	JSOOn	JGS	JCO	JSOP	JSAIO	JRVS	JNOS	Total		
Women	1	1	3	5	2	7	59	4	1	9	3	209		
Men	9	4	19	31	15	58	105	17	11	47	38	951		
Women (%)	10.0	20.0	13.6	13.9	11.8	10.8	36.0	19.0	8.3	16.1	7.3	18.0		

JAOI = Japanese Association for Ocular Infection, JASA/JAPO = Japanese Association for Strabismus and Amblyopia/Japanese Association for Pediatric Ophthalmology, JCC = Japan Cornea Conference, JCLS = Japan Contact Lens Society, JCO = Japan Clinical Ophthalmology, JGS = Japan Glaucoma Society, JIPS = Japan Imaging and Perimetry Society, JMS = Japan Myopia Society, JNOS = The Japanese Neuro-Ophthalmology Society, JOIS = Japanese Ocular Inflammation Society, JOS = Japan Ophthalmological Society, JRVS = Japanese Retina and Vitreous Society, JSAIO = Japanese Society of Artificial Intelligence in Ophthalmology, JSCR = Japanese Society for Cataract Research, JSCRS = Japanese Society of Cataract and Refractive Surgery, JSLRR = Japan Society of Low-vision Research and Rehabilitation, JSLT = Japanese Society of Lacrimal Passage and Tear Dynamics, JSOC = Japanese Society for Ocular Circulation, JSOD = The Japanese Society of Ophthalmic Diabetology, JSOOn = Japanese Society of Ocular Oncology, JSOOp = Japanese Society of Ophthalmological Optics, JSOP = Japanese Society for Ocular Pharmacology, JSOPRS = Japanese Society of Ophthalmic Plastic and Reconstructive Surgery, JSOS = Japanese Society of Ophthalmic Surgery.

* Joint lecture of JCLS, JOIS, JAOI, and JSLT.

**Figure 1.**
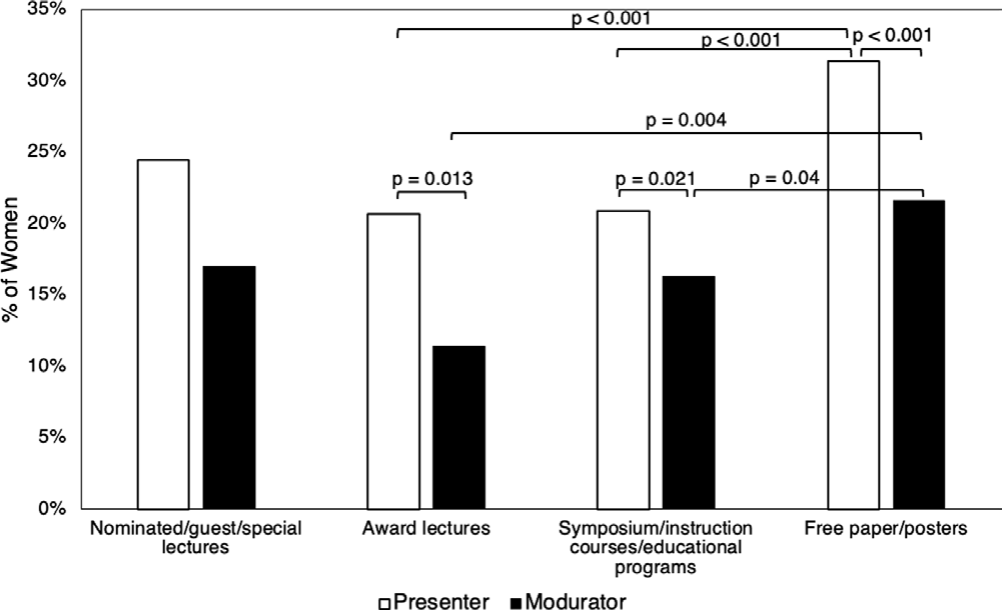
The percentage of the female first presenters and moderators among the nominated/guest/special lectures, the award lectures, the symposium/instruction courses/educational lectures, and the free paper/poster. The percentage of women is highest for the free paper/poster, both among the first presenters and the moderators. The percentage decreases according to the level of the lectures ascends, however; it increases again in the nominated/guest/special lectures.

When we further classified the lectures into 4 categories; nominated/guest/special lectures, award lectures, symposium/instruction/educational lectures, and free paper/posters, female representation among first presenters comprised 24.5%, 20.7%, 20.9%, and 31.4%, and among moderators, 17.0%, 11.4%, 16.3%, and 21.6%, respectively (Fig. 1, Table 3). A significantly higher proportion of female presenters was observed in the free paper/poster category compared to other categories (*P* < .001 vs award lectures and symposia/instructional/educational lectures for first presenters; *P* = .040 and 0.004 vs symposia/instructional/educational lectures and award lectures for moderators, respectively).

**Table 3:** Gender gaps in the moderator and the first presenters in 4-type categorized lectures.

		Moderators						First presenters					
		Japanese		Non-Japanese		Total (% of women)		Japanese		Non-Japanese		Total (% of women)	
Nominated/guest/special lectures	Women	8	100.0%	0	0.0%	8	(17.0%)	6	50.0%	6	50.0%	12	(24.5%)
	Men	39	100.0%	0	0.0%	39		28	75.7%	9	24.3%	37	
Award lectures	Women	16	94.1%	1	5.9%	17	(11.4%)	58	100.0%	0	0.0%	58	(20.7%)
	Men	132	100.0%	0	0.0%	132		219	98.6%	3	1.4%	222	
Symposium/instruction courses/ educational programs	Women	75	98.7%	1	1.3%	76	(16.3%)	270	98.9%	3	1.1%	273	(20.9%)
	Men	389	100.0%	0	0.0%	389		1017	98.6%	14	1.4%	1031	
Free paper/posters	Women	105	97.2%	5	2.8%	108	(21.6%)	768	99.4%	5	0.6%	773	(31.4%)
	Men	391	100%	0	0.0%	391		1677	99.1%	15	0.9%	1692	

**Figure 2.**
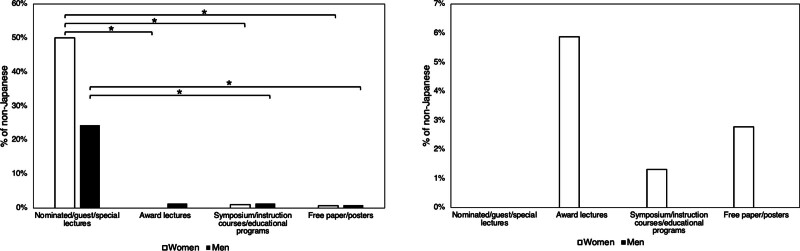
The percentage of the non-Japanese first presenters and moderators among the nominated/guest/special lectures, the award lectures, the symposium/instruction courses/educational lectures, and the free paper/poster. Left; the percentage of the non-Japanese first presenters is high in the nominated/guest/special lectures for both genders, however, quite low among the other 3 types of lectures. Right; the non-Japanese moderators were quite few (below 6.0%) in all types of the lectures (*, *P* < .001).

The proportion of non-Japanese individuals was 0.0% among male moderators, 2.4% among female moderators, 1.4% among male first presenters, and 1.3% among female first presenters. Of the 4 lecture categories, only the nominated/guest/special lectures demonstrated a significantly higher proportion of non-Japanese first presenters (24.3% in men and 50.0% in women; *P* < .001 between the nominated/guest/special lectures and the other 3 lectures for the women, and the nominated/guest/special lectures and symposium/instruction/educational lectures and the free papers/posters for men, respectively). The proportion of non-Japanese male moderators was 0%. Conversely, the proportion of non-Japanese female moderators was 5.9% in award lectures, 1.3% in symposium/instruction courses/educational programs, and 2.8% in the free paper/poster category (Fig. 2; Table 3). However, the role of the non-Japanese female moderator was fulfilled solely by a single female professor.

## 4. Discussion

This study found that women served as the first presenter or moderator in 25.2% of domestic Japanese congresses. Among them, women comprised 27.2% of first presenters and 18.0% of moderators. Female participation was greatest among free paper/poster presentations, followed by nominated/guest/special lectures, symposia/instructional/educational lectures, and finally, award lectures, for both presenters and moderators. The representation of non-Japanese individuals as first presenters was 1.4% in men and 1.3% in women, contrasting with 0.0% and 2.4% for male and female moderators. In contrast, nominated/guest/special lecturers showed a 24.5% and 50.0% representation, respectively.

A significantly lower proportion of women were present among first presenters and moderators in Japanese domestic congresses than the 42.2% and 37.3% of female ophthalmologists reported in hospitals and clinics, respectively, in 2022 by Japan’s Ministry of Health, Labour and Welfare (https://www.mhlw.go.jp/toukei/saikin/hw/ishi/22/index.html). These results indicated a disparity in academic output between male and female ophthalmologists in Japan, with the latter exhibiting lower levels of activity. These facts may indicate that female ophthalmologists may have fewer opportunities for academic contributions alongside their clinical work, despite possessing the requisite ideas and motivation, particularly when balancing family responsibilities. Our prior research examining gender disparity amongst Japanese ophthalmologists revealed female ophthalmologists shoulder primary familial responsibilities, consequently limiting their professional work hours relative to their male counterparts.^[24]^ The findings seen in the present study may support our current hypothesis.

Among the subspecialties, a higher proportion of female first presenters was observed at the following congresses: JASA/JAPO, JSLRR, JIPS, JCC, and the Japanese Society of Ophthalmic Plastic and Reconstructive Surgery. Within these subspecialties, patients typically present with chronic conditions, resulting in shorter surgical durations and fewer emergency procedures required. Conversely, congresses exhibiting a lower proportion of female presenters included Japanese Society of Ocular Oncology, followed by JSLT, Japanese Society of Ophthalmic Diabetology, Japanese Society of Ophthalmic Surgery, and JSCRS. Ophthalmologists participating in these congresses are more likely to undertake complex surgical procedures with a higher risk of severe complications or emergencies.

The female ophthalmologists may be less likely to pursue or may be actively excluded from subspecialties involving extensive or emergency surgery. In Japan, traditional gender roles persist in both professional and domestic spheres, disproportionately relegating female ophthalmologists to secondary professional roles and primary childcare responsibilities. The established pattern of extensive working hours underscores this tendency. Consequently, extended surgical procedures, including those necessitated by emergencies, like vitrectomies or glaucoma surgeries, are predominantly undertaken by male surgeons. Female ophthalmologists, conversely, are more likely to focus on medical treatments or shorter surgical interventions.

Our comparison of first presenters and moderators revealed a significantly lower representation of women among moderators. Notably, free paper/poster sessions exhibited the highest proportion of female first presenters across all session types. A similar tendency was reported previously by some authors from Canada and the USA.^[25–28]^ In Japan, ophthalmology residents must present at least 2 papers or posters at medical congresses and publish at least 1 paper in order to obtain a registered diploma of ophthalmology. These presentations could be case reports or the outcomes of simple observational studies. However, participation as a moderator, presenter, or panelist in advanced lectures necessitates a comprehensive presentation of research findings on consistent topics. A lack of cumulative research and publication achievements may limit their opportunities to present and/or moderate at high-profile congress lectures. The discrepancy between the incidence of the female presenters and moderators among the session types may again indicate that the female ophthalmologists abandon the opportunities for academic contributions alongside their clinical work after they obtain a diploma of ophthalmology because of private/family issues.

There are also numerous previous publications regarding the issue of the gender gap in authorship. Among them, Lagsi et al^[29]^ and Sidhu et al^[30]^ reported that the prevalence of both female first- and senior authors increased from 1970 to 2004 in the United States and the United Kingdom, respectively. However, the prevalence of senior authors consistently remained lower than that of first female authors. They supposed that it was because of a shortage of women in the academic pipeline, constraints imposed by traditional gender roles, manifestations of sexism within the medical environment, and a lack of effective mentors and institutional support for female faculty with children. Mueller et al^[31]^ investigated the gender gap in publications within US academic surgery and reported that the differences were larger in the rank of associate professor and full professor than in post-fellowship years. These results by previous authors were consistent with our present results and may suggest some solutions for us.

Another significant finding of this investigation was the absence of foreign presenters and moderators. A low proportion of non-Japanese first presenters was observed (1.4% for men and 1.3% for women), with a lower proportion in the moderator role (0.0% for men, 2.4% for women). This is likely attributable to the use of Japanese as the primary language in most Japanese domestic congresses. The only exception was the presenter’s role in the nominated/guest/special lectures which revealed a non-Japanese speaker representation of 24.3% among male presenters and 50.0% among female presenters. Japanese domestic ophthalmology congresses frequently feature prominent foreign clinicians and researchers as keynote speakers, a disparity reflected in the underrepresentation of women in these key roles, possibly indicating insufficient support for female researchers within ophthalmological societies.

A marked increase in female students in Japanese medical schools suggests women will soon constitute a majority of ophthalmologists. If we continue this tendency, the number of ophthalmologists, who can perform complex surgical procedures with a higher risk of severe complications or emergencies, will decrease in the near future, resulting in the shortage of ophthalmologists. To resolve these issues, we propose a review of postgraduate ophthalmology training to better support female surgeons in enhancing their surgical and academic performance. Thorough evaluations of workplace operations and scheduling, mentorship for junior researchers, and proactive advancement of women into academic leadership roles, including leadership training, are necessary. Simultaneously, using English as a primary language in the domestic congresses may affect the participation of more foreign presenters at the Japanese congresses, presumably resulting in defying the stereotypes and expanding our horizons.

A limitation of this study is the potential inaccuracy of gender and nationality data, due to reliance on web and abstract-based sources. Nevertheless, in the majority of cases, it was possible to ascertain the gender and nationality of a Japanese person based on their name. In fact, the ambiguous instances constituted a mere 1.5% and thus likely had a minimal impact on the survey results.

In conclusion, this study revealed that the Japanese domestic congresses in ophthalmology are Japanese male-dominated. The improvement of this situation requires a comprehensive review of the working environment and significant support for the advancement of young female ophthalmologists and researchers.

## Acknowledgments

The authors wish to thank Dr Maiko Yamashita, Department of Ophthalmology, Tsukuba University Hospital, for her help in collecting the data.

## Author contributions

**Conceptualization:** Hiromi Onouchi, Akemi Iwasaki, Mariko Itakura, Yoichi Manabe, Naoko Kato.

**Data curation:** Hiromi Onouchi, Akemi Iwasaki, Yuka Morita, Mariko Itakura, Keiko Kunimi, Naoko Kato.

**Formal analysis:** Hiromi Onouchi, Yuka Morita, Keiko Kunimi.

**Investigation:** Hiromi Onouchi, Akemi Iwasaki, Yuka Morita, Mariko Itakura, Keiko Kunimi, Yoichi Manabe, Naoko Kato.

**Methodology:** Hiromi Onouchi, Yoichi Manabe, Naoko Kato.

**Project administration:** Naoko Kato.

**Resources:** Akemi Iwasaki, Yuka Morita.

**Supervision:** Yoichi Manabe, Naoko Kato.

**Validation:** Keiko Kunimi.

**Writing – original draft:** Hiromi Onouchi, Naoko Kato.

**Writing – review & editing:** Akemi Iwasaki, Yuka Morita, Mariko Itakura, Keiko Kunimi, Yoichi Manabe.
